# Dynamic interactions of the cortical networks during thought suppression

**DOI:** 10.1002/brb3.503

**Published:** 2016-06-16

**Authors:** Toshihiko Aso, Kazuo Nishimura, Takashi Kiyonaka, Takaaki Aoki, Michiyo Inagawa, Masao Matsuhashi, Yoshikazu Tobinaga, Hidenao Fukuyama

**Affiliations:** ^1^ Human Brain Research CenterKyoto University Graduate School of MedicineKyotoJapan; ^2^ Institute of Economic ResearchKyoto UniversityKyotoJapan; ^3^ Kyoto University Graduate School of EducationKyotoJapan; ^4^ Elegaphy, Inc.OtsuJapan

**Keywords:** Executive control network, functional MRI, independent component analysis, inhibitory function, visual imagery

## Abstract

**Objectives:**

Thought suppression has spurred extensive research in clinical and preclinical fields, particularly with regard to the paradoxical aspects of this behavior. However, the involvement of the brain's inhibitory system in the dynamics underlying the continuous effort to suppress thoughts has yet to be clarified. This study aims to provide a unified perspective for the volitional suppression of internal events incorporating the current understanding of the brain's inhibitory system.

**Materials and Methods:**

Twenty healthy volunteers underwent functional magnetic resonance imaging while they performed thought suppression blocks alternating with visual imagery blocks. The whole dataset was decomposed by group‐independent component analysis into 30 components. After discarding noise components, the 20 valid components were subjected to further analysis of their temporal properties including task‐relatedness and between‐component residual correlation.

**Results:**

Combining a long task period and a data‐driven approach, we observed a right‐side‐dominant, lateral frontoparietal network to be strongly suppression related. This network exhibited increased fluctuation during suppression, which is compatible with the well‐known difficulty of suppression maintenance.

**Conclusions:**

Between‐network correlation provided further insight into the coordinated engagement of the executive control and dorsal attention networks, as well as the reciprocal activation of imagery‐related components, thus revealing neural substrates associated with the rivalry between intrusive thoughts and the suppression process.

## Introduction

The psychological mechanism of thought suppression has attracted continued interest because of the paradoxical or “ironic” difficulty involved in the prohibition of a particular thought (Wegner et al. 1987). A common experience is of thoughts or images that tend to surface despite (or rather because of) our effort to suppress them, with these thoughts becoming more pronounced and even disabling in pathological states such as obsessive–compulsive disorder (OCD) (Rassin et al. 2000; Najmi et al. 2009; Magee et al. 2012). Hence, it is clinically relevant to understand the mechanism underlying the competition between intrusive thoughts and efforts to cope with them. The present functional MRI (fMRI) study aims to gain insight into this inherently subjective experience using a recently introduced approach.

There are two lines of brain mapping studies related to this phenomenon: one approach focuses on rivalry or competition, whereas the other emphasizes the suppression/inhibition process per se. Many studies on the neural correlates of “thought suppression” have targeted these competing processes, typically using a long suppression period of up to 120 sec (Wyland et al. 2003; Gillath et al. 2005; Kalisch et al. 2006; Mitchell et al. 2007). Wyland and others found anterior cingulate cortex (ACC) activation during thought suppression in comparison with free‐thought conditions. These authors also reported that bilateral insula were additionally recruited during the complete suppression of any thought, that is, clearing of the mind. Gillath and others reported two medial prefrontal regions, including the ACC, as suppression related, whereas Kalisch and others observed left lateral prefrontal activation by suppression of thoughts or feelings related to anxiety or shock. Mitchell and others reported the right dorsolateral prefrontal cortex (DLPFC) and the ACC to be responsible for sustained and transient suppression, respectively, under the assumption that transient control occurred when the participant noticed intrusive thoughts and pressed a button. Although the inferior frontal gyrus (IFG) and supramarginal gyri (SMG) were also detected, the authors did not incorporate these regions into their model. Although this group of studies individually provides clues regarding the brain regions involved in thought suppression, brain activity appears to be context dependent and difficult to aggregate into a cohesive whole. Indeed, a common subject of interest shared by these studies is task difficulty, especially in the study by Mitchell et al., in which participants were asked to press a button when they accidentally failed to suppress thought. Accordingly, the relatively constant detection of the ACC has been interpreted to reflect the nonspecific cognitive demand or effort of suppression (Magee et al. 2012).

The other group of studies typically employ brief trial durations, and the term “thought suppression” is not necessarily used for the target process, as they focus on the inhibition process only (Garavan et al. 1999; Butler and James 2010; Benoit and Anderson 2012; Banich et al. 2015; Depue et al. 2015). Despite varying task settings, the reports closely agree with regard to the involvement of the anterior part of the lateral prefrontal cortex (LPFC) often with the lateral parietal regions with right‐side dominancy. A recent prominent study has demonstrated that the suppression of motor, cognitive, and emotional responses all share a set of frontal and parietal regions (Depue et al. 2015). Another study revealed differences among strategies used to suppress unwanted thought and confirmed that direct suppression involves a part of the right frontoparietal network (FPN) (Benoit and Anderson 2012). The relevance of the right LPFC in the inhibitory process has also been noted by a body of clinical studies on OCD as well as attention‐deficit hyperactivity disorder (Depue et al. 2010; Rubia et al. 2010; Munakata et al. 2011).

Thus, the neural substrates of inhibitory processes have become established at least for transient thought suppression. Why, then, are such activities not always found during a longer period of thought suppression? One reasonable explanation is that paradoxical difficulty itself introduces considerable fluctuations in the inhibitory activity. As depicted in the original work by Wegner et al. (1987), the participants report frequent intrusive thoughts because the essentially challenging part is maintenance, or making a specified subject *never* surface. Such a random, untraceable fluctuation of the task performance would diminish the sensitivity of model‐driven analysis approaches through inefficient modeling of fMRI response.

Another issue that may have lead to insufficiently convergent evidence for the neural correlates of thought suppression is the contamination of other cognitive processes. Higher brain functions are known to be task sensitive and it has already been argued that the target of inhibitory process should vary depending on the experimental settings (Depue 2012). As noted above, the ACC activity repeatedly observed during suppression has been interpreted to reflect cognitive components not strictly specific to inhibition, including effort, conflict/competition, and control. Given that transient inhibition per se can be a passive and even easy task, these task‐related activities may affect the results. For instance, recent advances have shown that keeping in mind a stimulus–response correspondence, or a task set, already requires continuous activity in a group of regions including the ACC (Dosenbach et al. 2006; Kennerley et al. 2009). Indeed, it is logically impossible for the participant to *completely* clear her mind while successfully continuing the experiment. Although relatively simple tasks were used in those earlier trials, there were typically more than two conditions in the experiments that the participants were required to switch between internally (Wyland et al. 2003; Gillath et al. 2005; Mitchell et al. 2007). Every cognitive/metacognitive component in the session structure, such as self‐monitoring by introspection, switching between multiple conditions, and even the error response (Ullsperger and von Cramon 2001), might affect the brain response.

In this study, we aimed to advance the understanding of this interesting phenomenon using a novel combination of simple task paradigm and data‐driven analysis approach. To minimize unwanted task load by additional cognitive/metacognitive components, we designed the experiment so that it involved only two conditions: visual imagery and thought suppression, or ceasing thoughts (Aoki et al. 2013). The participants were asked to visualize a famous architectural work during the imagery blocks, separated by suppression blocks during which they were instructed to avoid thinking of anything insofar as it was possible. The rationales behind this simple setting were as follows: (1) to introduce another condition for baseline would not only increase the task set‐related activity but also make the participants actively monitor their own mind throughout the experiment and (2) that unconstrained rest is difficult to model unambiguously (Stark and Squire 2001; Morcom and Fletcher 2007; Spreng 2012).

This simplified task setting was combined with an independent component analysis (ICA) based, data‐driven method for decomposition and classification of the brain activity by task‐related variation. Due to the implicit nature of the task, measurement of the suppression performance has required online self‐reporting that should again confound the brain activity. A recent study dealt with this issue head‐on by referring to the fMRI signal as a biomarker for successful inhibition (Banich et al. 2015). If applied with decent caution, this “reverse correlation” approach would extend the use of functional imaging for mapping brain functions in a real‐life setting (Friston and Henson 2006; Poldrack 2006). Thus, we employed a combination of ICA decomposition and post hoc analysis approach (Bartels and Zeki 2004; Hasson 2004). This type of study requires interpretation of *every* component based on a wide range of earlier functional mapping literatures to unambiguously relate activity components with cognitive processes. In return, this procedure allows both identification of task‐related activities and evaluation of their within‐task fluctuation such as reciprocal or coping behaviors. Based on the temporal dynamics of the task‐related components, we present a comprehensive perspective of the classical thought suppression process in the context of the brain's inhibitory mechanism.

## Materials and Methods

### Subjects and experimental procedures

From a group of 99 university students who were naïve to thought suppression phenomenon, we recruited 20 right‐handed individuals (19 Japanese and one Chinese, five females; mean age = 21.5 ± 0.9, range = 20–23 years old) with no history of neurological problems. Informed consent was obtained in a manner approved by the university medical school IRB. Before the fMRI session, participants received a brief interview concerning the subjective evaluation of their ability to suppress thoughts. First, the participants were asked to think about nothing for 1 min, after which they answered the question “Did you successfully suppress your thoughts?” with a yes or no.

Inside the MRI scanner, subjects viewed a white fixation point that flashed for 2 sec every 24 sec to cue the progression to the next block. The flash of the fixation point was green except at the beginning of the first Imagery block, after which two “Imagery” blocks and two “Suppression” blocks followed. For the “Imagery” condition, participants were instructed to picture two famous architectural structures from Japan in the order: the Kinkaku in Rokuon‐ji temple, commonly known as “Kinkaku‐ji,” for the first 24 sec and the Japanese Diet Building for the latter 24 sec. The instruction was given only verbally, and no actual picture was presented. For the “Suppression” condition, we asked them to “avoid insofar as possible thinking of anything,” only implying imagery suppression. This instruction was chosen on the basis of instruction simplicity, thereby minimizing metacognition. Each participant went through two 408 sec runs containing four block pairs.

### Image acquisition

A Siemens (Erlangen, Germany) Trio 3T scanner with an 8‐channel phased‐array head coil was used to obtain structural and functional images. For fMRI, T2*‐weighted echo‐planar images were acquired with following parameters: slice thickness = 4 mm; 40 axial slices; repetition time (TR) = 2.0 sec; echo time (TE) = 30 ms; flip angle = 90°; matrix size = 64 × 64; field of view (FOV) = 192 × 192 mm. Three‐dimensional T1‐weighted image acquisition followed with following parameters: TR = 1630 ms, TE = 4.38 ms, inversion time = 990 ms, FOV = 240 mm, voxel size = 0.94 × 0.94 × 0.95 mm, 8° flip angle, 130 Hz bandwidth.

### Data processing

The images were preprocessed using SPM (SPM8, RRID:SCR_007037; Wellcome Department of Cognitive Neurology, London, UK) on MATLAB. This process included interscan slice timing correction, three‐dimensional motion correction, and spatial normalization to the Montreal Neurological Institute template (Mazziotta et al. 1995). The functional images were warped according to these linear and nonlinear transformations and then resliced and smoothed using an isotropic Gaussian filter with a kernel width of 8 mm. Four image volumes at the beginning of each session were discarded to remove initial deflection, leaving 200 volumes per session for the following analyses.

### Group ICA

Independent component analysis was carried out using the Group ICA of fMRI Toolbox, RRID:SCR_001953 (http://icatb.sourceforge.net) (Calhoun et al. 2001). With no information on the task structure, the fMRI data were subjected to the ICASSO pipeline in which the ICA optimization was repeatedly run 10 times both bootstrapping (resampling) the data and randomizing initial conditions (Himberg and Hyvarinen 2003). During these iterations, a repeated occurrence of similar components indicated the robustness of each estimated component as a strong cluster to survive in a “similarity graph.” The clustering was performed with hierarchical agglomerative clustering using the average linkage criterion. We chose the Infomax algorithm with a predetermined number of components, or model order, of 30. Choosing this value depending on the goal of the studies is a widely accepted strategy based on the findings of earlier studies (Calhoun and Ph 2009; Abou‐Elseoud et al. 2010; Cole et al. 2010; Smith et al. 2012). We chose the value of 30 as a good compromise between insufficient decomposition (too small) and excessive splitting of the major cortical networks (too large) (Aso and Fukuyama 2015).

### Component classification

To operationally label independent components (ICs) that represent motion‐related or other physiological/mechanical artifacts, the time course of each component in each subject was Fourier transformed. Then, we calculated low‐frequency power ratio (LFPR) as the ratio of the sum of spectral power between 0.003 and 0.10 Hz to the sum between 0.15 and 0.25 Hz (Allen et al. 2011; Yu et al. 2013). In line with a previous report (Allen et al. 2011), LFPR values <3 were only observed in clearly artifactual components, and values <3.5 were considered indicative of artifactual origin. We kept this procedure conservative by leaving some of these suspicious ICs in the subsequent analysis. By sorting the 30 group ICA components by LFPR, the first six clearly originated from eyeball movements, cerebrospinal fluid in the ventricles, and other factors and were labeled as artifacts (Fig. S1). An additional five ICs were also discarded due to their excessive involvement in nonbrain regions as indicated by low signal level in the original images (Smith et al. 2012). The nonartifactual components were thresholded at *z* > 2 and identified as known cortical networks by template matching (http://fsl.fmrib.ox.ac.uk/analysis/brainmap+rsns/) (Smith et al. 2009).

#### Fluctuation index

Motivated by the interest in within‐task random change in the brain state, we defined “fluctuation index”. This is an fMRI adaptation of Fano factor (Qi and Constantinidis 2012) which measures intertrial variability of the response. After bandpass filtering at 0.005–0.1 Hz, a component's time course during one of the task conditions was extracted and grand averaged over trials and subjects. This pooled response was then subtracted from the original, raw responses, from which standard deviation over time was calculated for each subject. By dividing this standard deviation by the grand‐averaged signal amplitude, fluctuation index was defined for each component from each participant. The index thus reflects the amount of random deviation from the typical response pattern of the component, normalized by the typical response magnitude. The fluctuation indices over all components, participants and tasks were compared to evaluate (1) the effect of task (Imagery or Suppression) and (2) their correlation with subjective ability to suppress thoughts.

#### Temporal correlation analysis

The residual temporal correlation across component time courses can be used to evaluate relationship between networks (Seeley et al. 2007; Smith et al. 2013). In this study, we sorted the ICs by their relationship with IC19, the Suppression‐related component (see Results). To capture the correlation in within‐task fluctuation, first, Pearson's correlation coefficient was calculated for each block after discarding the initial four volumes (=8 sec) and detrending on the bandpass‐filtered session time course. The correlation coefficient was converted to Fisher's *Z* and pooled over trials. For both Imagery and Suppression blocks, one‐sample *t*‐test over participant were calculated to estimate the polarity of correlation in general population. A paired *t*‐test was also conducted to search for change in correlation by task, but no pairs under investigation showed significance at the *P* = 0.05 level, meaning that no pair exhibited a significant change in correlation with polarity during the two tasks. Therefore, we labeled each IC as either positively or negatively correlated with IC19 only if it presented significant nonzero correlation during both of the tasks.

### Statistical analysis

#### Task‐relatedness

Using the individual component time courses back‐projected from the group ICA (Calhoun et al. 2008b), we conducted two types of group analysis. The main analysis (Analysis 1), which is more conservative and allows inference to the general population, consisted of individual multiple linear regression followed by a one‐sample *t*‐test of the parameter estimates over participants (Meda et al. 2009; Zhang and Li 2012). The first‐level multiple regression involved the modeling of each IC time course by four regressors, corresponding to the two consecutive imagery blocks and two rest blocks. Block transitions cued by the color of the fixation point were also designed as a set of events with zero duration. These regressors were created by the convolution of the temporal structure with the canonical hemodynamic response to model the IC time course as follows:

y=Xβ+ε

where ***X*** is the matrix of the eight regressors and *β* is the set of estimated parameters. Thus, the task relevance of every IC from every subject was measured by the difference between the *β* values for Imagery and Suppression blocks. For each IC, a one‐sample *t*‐test against zero was computed on the *β* weight differences to find networks that were significantly associated with the task phase.

For the second analysis (Analysis 2), to sort ICs by their relative, albeit marginal, task engagement, we first averaged the mean individual IC time courses over participant and trial. An independent two‐sample *t*‐test was performed on the mean Imagery/Suppression block signals to detect the component with higher activity on average during either of the conditions.

### GLM analysis

In addition to the ICA, a general linear model (GLM) analysis was performed using SPM8 in order to confirm the technical advantage of the data‐driven approach. Each run was incorporated into the design matrix with a regressor to model the signal change between the two conditions and eight regressors of no interest for (1) the onset of either the Imagery or Suppression condition that occurred every 24 sec and (2) six head motion parameters. Similar to the regression analysis described above, these task‐related regressors were created by convolving the boxcar/impulse functions with the canonical hemodynamic response. After individual GLM, the results were fed into a second‐level analysis to perform a random‐effects model group analysis. The resulting T‐maps were thresholded at *P* = 0.05 and corrected for multiple comparisons in space.

## Results

### Group ICA

#### Suppression‐related ICs

**Figure 1:**
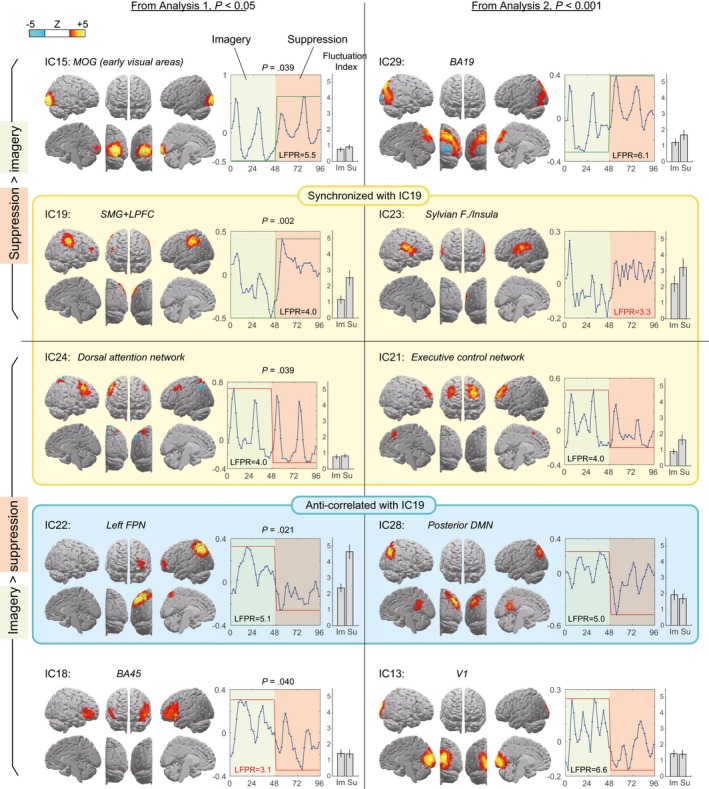
Components that survived the task‐relatedness tests by the main (conservative) group analysis (Analysis 1, left column) and the pooled time course analysis (Analysis 2, right column). *Z*‐score maps are thresholded at *z* = ±2. The four ICs on the top were Suppression related, whereas the other six were Imagery related. Yellow/blue background indicates ICs that are positively/negatively correlated with the IC19 in temporal fluctuation, respectively. Time courses were pooled over trials and participants from the alternating pair of Imagery (green background) and Suppression (red background) blocks. Next to the pooled time course, a bar graph presents the within‐block fluctuation index. Error bars indicate standard deviation over subjects. The differences in fluctuation index between the two tasks were significant for all components shown here except for IC18 and IC24 (*P* < 0.05). LFPR, low‐frequency power ratio; MOG, middle occipital gyrus; BA, Brodmann's area; FPN, frontoparietal network.

Of the 19 valid ICs, only five survived the main statistical analysis of task relevance either positively or negatively (Fig. 1, left column). The most significantly task‐related component was IC19: the right‐side‐dominant bilateral SMG + LPFC (BA46; peak at [46, 44, 14]). Another significantly Suppression‐related component was IC15, the bilateral occipital cortices centered on the middle occipital gyri. The secondary statistical analysis on the IC time course aimed to measure relative task relevance detected two additional ICs (Fig. 1, right column): the higher visual cortices (IC29) and the Sylvian fissure/insular (IC23) components. However, the IC23 seemed to suffer considerable noise contribution judging from the location of the *z*‐score peak in the cerebrospinal fluid (CSF) voxels (Montreal Neurological Institute stereotaxic space coordinate: [−58, 20, 14]) and the LFPR <3.5 (Allen et al. 2011).

#### Imagery‐related ICs

The left FPN with dominant involvement of the intraparietal sulcus (IC22) showed the strongest Imagery engagement, followed by the dorsal attention network (DAN) (Corbetta et al. 1998) (IC24) and left‐side‐dominant lateral prefrontal regions peaking at Brodmann's area (BA) 45 (IC18). The secondary analysis detected the posterior default mode network (DMN) (IC28), primary visual cortices (IC13), and executive control network (ECN, IC21) composed of the bilateral anterior dorsolateral and medial prefrontal cortices (MPF) (Duncan and Owen 2000; Smith et al. 2009) as Imagery related. Among these components, the IC18 had a low LFPR score, which again indicates some noise contamination.

#### Other components

**Figure 2:**
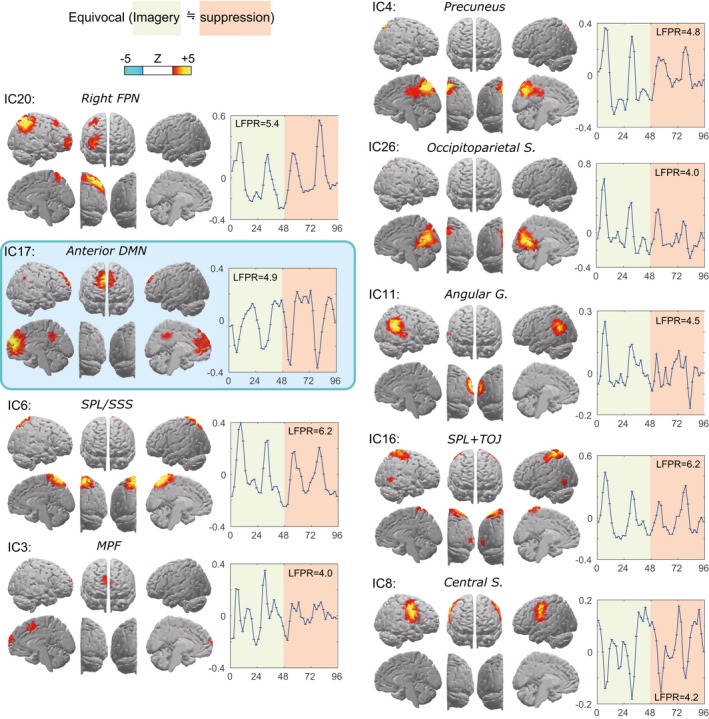
The components whose task relevance did not reach significance, presented in the same convention as in Figure 1. LFPR, low‐frequency power ratio; S, sulci; G, gyri; FPN, frontoparietal network; DMN, default mode network; SPL, superior parietal lobules; SSS, superior sagittal sinus; TOJ, temporo‐occipital junction; MPF, medial prefrontal cortices.

Nine components with equivocal task engagement are shown in Figure 2. A time course of the right FPN (IC20) was markedly different from its left counterpart (Smith et al. 2009). Only the anterior DMN exhibited a negatively correlated fluctuation with the IC19, which replicates its posterior counterpart (Fig. 1). The angular gyri were detected as a separate component supporting the specific involvement of the SMG in thought suppression. IC6 covered the superior parietal lobule (SPL) and exhibited a similar time course to the DAN component (IC24), but its midline location peaked at the superior sagittal sinus with its lateral lacunae indicating a significant inclusion of draining veins. The SPL appeared to be better represented by IC16 with separate peaks in the bilateral SPLs. This area includes the temporo‐occipital junction, where the human area MT+ is known to reside and coactivation with the SPL has been reported by visual attention tasks (Gitelman et al. 1999). IC3 included two medial prefrontal regions: frontopolar and dorsal regions anterior to the presupplementary motor area. These regions have been related to higher cognitive function such as metacognition, recognition of error, conflict and decision, all of which may have occurred in the experiment but with no detectable task‐related change in the present setting (Rushworth and Behrens 2008; Tsujimoto et al. 2010; Desmet et al. 2011). Finally, IC8 represented bilateral central sulci, primary somatosensory, and motor regions for the upper body parts (Laird et al. 2011).

#### Within‐condition fluctuation

Interestingly, the fluctuation index (Fig. 1, bar graphs), which is a measure of both intra‐ and intertrial random variation over the time course, significantly changed between tasks in all the components except for IC24 and IC18 (*P* < 0.05 by Student's *t*‐test on log‐converted values). The two most task‐engaged components, IC22 and IC19, presented twofold higher fluctuation during Suppression compared with Imagery. Only the posterior DMN component (IC28) showed decreased fluctuation during Suppression.

Seven out of 19 participants reported that they could successfully clear their mind during the 1‐min trial before the fMRI session. There was only one component, IC21 representing the ECN, that showed statistically significant correlation of task‐related fluctuation change with this subjective ability of thought suppression (Table 1). Participants who reported that they could suppress the thought had a higher positive task‐dependent fluctuation index, meaning that the network fluctuated selectively more for Suppression (*P* = 0.035, log‐converted *t*‐test). Refer to Figure S2 for the task‐fluctuation relationships of the other components.

**Table 1:** Statistical results from all valid components

Task relevance			Analysis 1 *P*‐value	Analysis 2 *P*‐value	Synchronization with IC19		Subjective report of 1‐min suppression (*P*)
					*P*	*Z*	
Supp	IC19	SMG + LPFC	0.00	<0.001			0.85
Img	IC22	Lt. FPN	0.02	<0.001	<0.01	−2.46	0.60
Supp	IC15	MOG	0.04	<0.001	0.81	−0.11	0.74
Img	IC18	BA45	0.04	<0.001	0.56	1.21	0.70
Img	IC24	DAN	0.04	<0.001	<0.01	3.79	0.45
Img	IC28	Post. DMN	0.05	<0.001	<0.01	−4.55	0.30
Supp	IC29	BA19	0.07	<0.001	0.30	0.00	0.42
Supp	IC23	Sylvian F.	0.10	<0.001	<0.01	2.86	0.39
–	IC4	Precuneus‐PCC	0.10	0.01	0.19	0.20	0.91
–	IC20	Rt. FPN	0.12	0.003	0.05	1.05	0.68
Img	IC13	V1	0.17	<0.001	0.58	−1.05	0.42
Img	IC21	ECN	0.22	<0.001	<0.01	4.70	0.04
–	IC26	Occipitoparietal S.	0.41	0.01	0.49	0.64	0.67
–	IC17	Ant. DMN	0.58	0.19	<0.01	−2.44	0.99
–	IC11	Angular G.	0.82	0.17	0.83	−0.29	0.63
–	IC6	SSS	0.89	0.06	0.03	1.26	0.74
–	IC16	SPL + TOJ	0.89	0.70	0.03	1.97	0.86
–	IC3	MPF	0.93	0.94	0.44	−1.25	0.67
–	IC8	Central S.	0.98	0.72	0.29	−0.47	0.11

SMG, supramarginal gyri; LPFC, lateral prefrontal cortex; FPN, frontoparietal network; MOG, middle occipital gyri; DAN, dorsal attention network; DMN, default mode network; BA, Brodmann's area; PCC, posterior cingulate cortices; V1, primary visual cortices; ECN, executive control network; SSS, superior sagittal sinus; SPL, superior parietal lobules; TOJ, temporo‐occipital junction; MPF, medial prefrontal cortices.

Shaded background indicates nonsignificance. Ten components survived in either of the two statistical tests. Among six components that showed a significant residual correlation with IC19, only IC17 (anterior DMN) failed to show task relevance. Only IC21 (ECN) was correlated with the subjective report of successful suppression.

#### Between‐network residual correlation

Motivated by the outstanding suppression relevance of the SMG + LPFC component, we measured the residual correlation of within‐block fluctuation between IC19 and all the other valid components (Seeley et al. 2007; Smith et al. 2013). As depicted in the Figure 1 and summarized in the Table 1, residual correlation classified the task‐relevant ICs based on their relationship with IC19 as positively correlated, negatively correlated, or equivocal. Among Imagery‐related components, the DAN and the ECN were positively correlated with the SMG + LPFC complex, whereas the left FPN and posterior DMN were negatively correlated, thus indicating reciprocal activation.

### Conventional GLM analysis

A random‐effects group analysis failed to detect significant task‐specific activity other than periodic response to block transition cues (at *P* = 0.05, family‐wise error corrected).

## Discussion

Overall, we considered task simplification, enabled by recently developed analysis approach, to be the primary novelty of this study, as it minimized confounders in identifying the essential brain regions among those previously noted. One of the major advantages of the ICA‐based model‐free method is that brain activity is decomposed into networks largely independent of the fMRI paradigm (Calhoun et al. 2008a). At the same time, because the large‐scale networks can split into two or more ICA components that are not necessarily preserved across studies, this type of analysis requires a evaluation for each of the components to obtain a rigorous argument on which any conclusions are based.

### The SMG + LPFC: suppression network

There was only a single, although prominent, Suppression‐related component representing the SMG + LPFC with slight right‐side dominancy (IC19). The current understanding of the inhibitory system allows us to interpret this as a case of activation by Suppression, rather than deactivation by Imagery (Swick et al. 2011; Zhang and Li 2012; Depue et al. 2015), given that thought suppression involves continuous inhibition. The time course with significant onset response followed by gradual decrease over the block may be partly due to high‐pass filtering, but also has some implications for the role of this system. In his original work, Wegner reported the highest occurrence of unwanted thought at the onset of the Suppression period, which was independent of the preceding task condition (Wegner et al. 1987). The peak activity at the onset may therefore indicate positive relationship of IC19 with the coping process of thought suppression, instead of successful inhibition.

**Figure 3:**
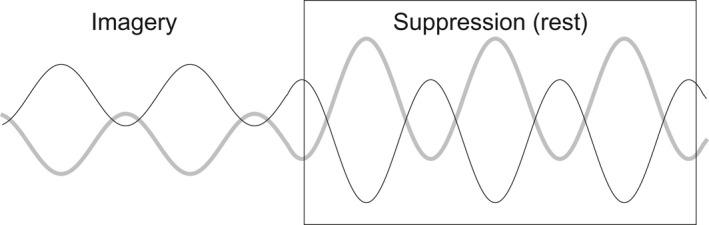
Schematic illustration of the two components representing the Imagery‐ (IC22, black line) and Suppression related (IC19, shaded line) systems. In addition to the task‐dependent baseline changes, both exhibited change in fluctuation which significantly increased during Suppression blocks. Residual temporal correlation between these two were constantly negative, indicating reciprocal activity. Due to the large fluctuation, these systems escaped detection by the GLM analysis.

The within‐task fluctuation of IC19 was negatively correlated with IC22, the most significant imagery‐related component. Figure 3 summarizes the findings from this analysis. This indicates reciprocal activity, further establishing the coupling of this component and inhibition. The remarkable increase in temporal fluctuation observed during Suppression is also consistent with the basic concept underlying thought suppression: inhibition is unstable. This pattern in turn provides an explanation for the failure of standard GLM analysis to define this activity at the population level, most likely as a result of the inefficiency of the boxcar model, which assumes constant activity. However, whether this fluctuation increase is specific to thought suppression requires further investigation because we chose a very simple instruction for the Suppression period, making the condition less constrained than the Imagery.

The *z*‐score map of IC19 peaked in the right anterior inferior parietal lobule (IPL), encompassing the SMG and extending to the angular gyri. Indeed, the IPL has been found to be constantly active during stop‐signal tasks (Aron and Poldrack 2006; Tabu et al. 2011), saccade suppression (Law et al. 1997; Pierrot‐Deseilligny et al. 2004), and inhibition in general (Nakata et al. 2008; Depue et al. 2015). In these earlier studies, including one by our group, interpretations of increased IPL activity, if any, could be summarized as nonspecific attention. Although the parietal lobe has not been assigned a primary role in inhibitory processes, the human IPL is at least thought to exemplify the topmost node in the hierarchy of postcentral brain regions, representing highly abstract thoughts (Hubbard et al. 2005; Coolidge and Overmann 2012). The significant asymmetry of and variation in functions within the SMG alone, such as language, arithmetic, mirror neuron system, and spatial cognition, suggest that almost all thought types are associated with this area (Hubbard et al. 2005; Hartwigsen et al. 2010). In addition, the SMG is highly sensitive to bistable perception (e.g., Necker cube or binocular rivalry), in which perception is fluctuating (Britz et al. 2008; Sterzer et al. 2009; Wilcke et al. 2009). Therefore, one possible interpretation of the major SMG recruitment observed in our findings would be that thought suppression involves the suspension of thinking. Because we instructed the participants to simply clear their minds as much as possible without specifying the subject to suppress, the participants must have employed direct suppression instead of substitution (Benoit and Anderson 2012).

This study does not provide information about the *causal* relationship of this activity with suppression; the network may be under control of some other system truly relevant to the task (e.g., the ECN. See below). Some earlier studies claim, however, central role of these regions in “generating” inhibition. In their extensive meta‐analytic work, Singh‐Curry and Husain (2009) argued that the right IPL is a crucial node in the frontoparietal system dedicated to flexibly allocating cognitive resources and not only to bottom‐up reorienting (Corbetta et al. 2008). Lesion studies also have provided clues that a competing plan of behavior is encoded in the IPL (Coulthard et al. 2008) and that saccade suppression requires intact IPL (Butler et al. 2006). Within the IPL, the differential involvement of the anterior and posterior parts in attention reorienting has been demonstrated by a transcranial magnetic stimulation study (Chambers et al. 2004). In line with earlier findings, this component's outstanding suppression‐relatedness and the relationship with other components in this study at least suggests an overlapping mechanism between attention reorientation and volitional internal suppression.

### Behavior of the major cortical networks during thought suppression and imagery

The residual correlation of IC21, the frontal component of the ECN, with IC19 has additional potential implications. This component was consistently synchronized with IC19 at two levels: within‐task fluctuation and between‐task changes in fluctuation. In addition, IC21 was the only component whose fluctuation change was linearly correlated with the subjective report of successful suppression. The human ECN was originally defined by Seeley et al. as a dissociable system from the saliency network by inclusion of a MPF region dorsal to the ACC, although there has been some variation in the nomenclature of subcomponents in the following reports (Seeley et al. 2007; Christoff et al. 2009; Smith et al. 2009; Wang et al. 2013). Prior to the functional connectivity analysis, a body of task‐based fMRI evidence has indicated the pivotal role of these regions in executive control (Braver et al. 2003; Dosenbach et al. 2007). In relation to the present finding about interindividual variability, there is another line of studies reporting relationship between sulcal structure in this region and frontal lobe functions including inhibition (e.g., measured by Stroop tasks) (Fornito et al. 2004; Borst et al. 2014; Cachia et al. 2014). Still the relationship between Suppression‐related fluctuation and subjective reports of suppression ability requires further investigation with more supporting behavioral measures. We should also note that the *z*‐score map of IC21 precisely covers frontal nodes of the network as originally described, but not in the parietal lobe. The observed tight synchronization might therefore suggest that IC19 and 21 are both subnetworks within the original human ECN.

IC24, covering the premotor cortex and SPL bilaterally, represents the so‐called DAN, where both implicit visual attention and eye movement are encoded. At the baseline level, IC24 was significantly Imagery related, in agreement with earlier works (Christoff et al. 2009). With respect to the functional coupling with IC19, the absence of a fluctuation change between task conditions may suggest only a minor causal relationship. Because the DAN strongly responded to every block transition (i.e., the blink of fixation point), this strong response may have contributed to this correlation even after the removal of the transition period. However, it is important to note that a conceivable response to block transition does not always result in significant correlation, as seen in the cases of IC15, IC18, and IC29.

Negatively correlated components with IC19 were both Imagery related (Fig. 1, blue background), meaning that their activities were consistently out of phase with IC19. Significant task relevance to Imagery was found in IC22, representing the left FPN with a major contribution from the posterior parietal cortex. There is accumulated evidence on the pivotal role of the left posterior parietal region in image generation (Christoff et al. 2009). By contrast, the right FPN (IC20, Fig. 2) had a completely different time course, with strong responses to each visual cue and slight signal decrement during Imagery. The right FPN also did not exhibit the same difference in fluctuation between the two conditions exhibited by its left counterpart, with a striking increase during the Suppression blocks. This strong negative correlation of the fluctuation between the left FPN and the Suppression‐related IC19 may represent a rivalry or competition that induces suppression instability. For future direction, the effect of instruction or task settings on the behavior of those networks would also be of considerable interest (Depue 2012).

The DMN was split into the anterior and posterior subnetworks, which is known to occur depending on the ICA parameters (Christoff et al. 2009). The posterior DMN (IC28) showed marginal Imagery‐related signal change, whereas no task relatedness was found in the anterior DMN (IC17). Although the FPN (IC22 and IC24) and DMN were initially recognized as task‐positive and ‐negative region groups (Christoff et al. 2009), these networks can work in synchrony depending on the task (Christoff et al. 2009). Because we used two famous architectural structures for the Imagery task, location‐ or navigation‐related activities may explain DMN involvement (Christoff et al. 2009).

### Visual and other networks

Another Suppression‐related component represented the caudal part of the occipital lobe centered on the middle occipital gyri (MOG) (IC15) with a marginal significance (*P* = 0.04). The BA19 (IC29) also exhibited increased activity by the pooled time course, in marked contrast with the Imagery‐related activity in the primary visual cortices (IC13). Despite consistent recruitment during visual perception, the occipital regions were often outside the regions activated by visual imagery (Christoff et al. 2009). Moreover, according to a detailed examination of how the early visual cortices are sometimes not activated by mental imagery, prediction of the visual cortical response in general is not straightforward (Christoff et al. 2009). Unfortunately, task‐related deactivation is rarely well documented; however, one study reported partial deactivation in the visual cortices during imagery (Christoff et al. 2009), and another recent study combining imagery with neurofeedback reported a paradoxical signal decrease in early visual cortices (Seeley et al. 2007; Smith et al. 2009; Wang et al. 2013), indicating general difficulty in mentally activating vision‐related regions. Thus, it seems that the neural activity during imagery in the regions represented by IC15 and IC29 are highly context dependent. Overall, the activity increase during the Suppression condition in the IC15, as well as the marginal task relevance exhibited by IC29, is compatible with earlier reports, although both requires further investigation for a thorough understanding.

IC23, another marginally Suppression‐related component, was the only one that significantly involved the insular cortices. This insular activation has been reported in both thought suppression and inhibition studies (Krmpotich et al. 2013; Kemmer et al. 2015; Lin et al. 2015; Rigon et al. 2015) but lacks an established interpretation. This area has been implicated in a wide variety of functions (Corbetta et al. 1998), including mental imagery (Mellet et al. 1996; Kosslyn and Thompson 2003; Ganis et al. 2004; Mazard et al. 2004; Sack et al. 2008). However, the local peaks of our IC23 were located in the Sylvian fissure, outside of the brain parenchyma, and the time course presented an excessive high‐frequency noise level (low LFPR) that is conceivable even after the grand averaging (Fig. 1). Given that a low LFPR is suggestive of the artifactual nature of an ICA component, our result at least indicates that interpretations must be made with caution (Formisano et al. 2002; Sack et al. 2008). A similar argument applies to another low‐LFPR component, IC18, which is Imagery‐related and centered at the BA45 bilaterally. This component covered the posterior IFG extending to the frontal operculum with left‐side dominancy, encompassing the frontal language area. Many studies have reported IFG involvement in mental imagery, despite the tasks being not necessarily language related (Smith et al. 2009; Tikka et al. 2012). However, besides the low LFPR, the component's location adjacent to the anterior insula, a region prone to respiration‐related artifacts (Shulman et al. 1997; Fox et al. 2005) may also suggest artifact contamination. The task relevance of these two components will remain inconclusive until further studies involving physiological monitoring examine their behavior.

## Conclusions

The brain signals decomposed by ICA indicated a dominant contribution of the SMG‐LPFC network in the inhibitory process during thought suppression, possibly under the control of the frontal nodes of the ECN. The negative correlation of fluctuation further suggests that the two Imagery‐related components are suppressed by this network. This inhibitory process is under the control of the ECN, the activity of which presumably reflects individual suppression ability. The efficiency of the present approach of using model‐free analysis on task‐loaded fMRI was obvious compared with the conventional model‐based approach.

The present findings also have clinical implications. According to a recent meta‐analysis study of brain structure in OCD, volume reduction in the right IPL is one of the most replicated findings among reports (Spreng 2012; Aso and Fukuyama 2015). There are even studies specifically showing SMG reduction (Epstein 2008; Spreng et al. 2009). Among these studies was a report on pure obsessive–compulsive patients presenting the reduction of white matter adjacent to the right SMG as the sole finding (Mellet et al. 1996; Ganis et al. 2004; Slotnick et al. 2005; Stokes et al. 2009; Bridge et al. 2012). A patient study with the present approach may connect these findings to illustrate a causal relationship and consequently the mechanism of the thought suppression difficulty in individuals suffering from obsession.

## Conflict of Interest

None declared.

## Supporting information


**Figure S1.** Components classified as artifacts by the rich fast frequency component (top six panels) and by the spatial distribution (bottom five panels). These components possibly originate from head motion, eyeball movements, cerebrospinal fluid, and draining veins/sinuses.

**Figure S2.** A, Ratio of fluctuation index between tasks. Error bars indicate standard deviation across participants. Fluctuation increased during Suppression reflecting less constrained nature of the condition relative to Imagery. B, Average variation index of the IC22 from the two groups of participants by the subjective performance of suppression. This was the only component that showed significance (*P* < 0.05, see Table 1).
